# Multimodality Imaging in Eosinophilic Myocarditis: A Rare Cause of Heart Failure

**DOI:** 10.3390/jcdd12080320

**Published:** 2025-08-21

**Authors:** Vincenzo Viccaro, Amabile Valotta, Elena Checcoli, Susanna Landi, Fabio Cattaneo, Andrea Milzi, Mattia Duchini, Giacomo Maria Viani, Alessandro Caretta, Susanne Schlossbauer, Antonio Landi, Laura Anna Leo, Giorgio Treglia, Giovanni Pedrazzini, Marco Valgimigli, Anna Giulia Pavon

**Affiliations:** 1 Department of Cardiology, Cardiocentro Ticino Institute, Ente Ospedaliero Cantonale, 6900 Lugano, Switzerland; vincenzo.viccaro95@gmail.com (V.V.); valotta.amabile@gmail.com (A.V.); elena.checcoli@eoc.ch (E.C.); susanna.landi@eoc.ch (S.L.); fabio.cattaneo@eoc.ch (F.C.); andrea.milzi@eoc.ch (A.M.); giacomo.viani@eoc.ch (G.M.V.); alessandro.caretta@eoc.ch (A.C.); susanne.schlossabauer@eoc.ch (S.S.); antonio.landi@eoc.ch (A.L.); lauraanna.leo@eoc.ch (L.A.L.); giovanni.pedrazzini@eoc.ch (G.P.); marco.valgimigli@eoc.ch (M.V.); 2 Faculty of Biomedical Sciences, Università Della Svizzera Italiana, 6900 Lugano, Switzerland; giorgio.treglia@eoc.ch; 3 Faculty of Biology and Medicine, University of Lausanne, 1015 Lausanne, Switzerland; 4 Division of Nuclear Medicine, Imaging Institute of Southern Switzerland, Ente Ospedaliero Cantonale, 6500 Bellinzona, Switzerland

**Keywords:** eosinophilic myocarditis, eosinophilic syndrome, acute heart failure, cardiogenic shock, cardiac magnetic resonance, multimodality imaging

## Abstract

Eosinophilic myocarditis (EM) is a rare and potentially fatal form of acute myocarditis. Currently, no validated diagnostic criteria exist, and definitive diagnosis relies on endomyocardial biopsy (EMB) not devoid of periprocedural complications. This review aims to explore how a multimodality imaging approach can support early diagnosis, reduce reliance on EMB, enable risk stratification and monitor the response to anti-inflammatory therapy. In particular, while echocardiography provides rapid and useful information in suspected EM, cardiac magnetic resonance (CMR) remains the non-invasive gold standard for diagnosis due to its ability to provide accurate tissue characterization. Moreover, positron emission tomography/computed tomography (PET/CT) and cardiac CT (CCT) may offer valuable insights, particularly when echocardiographic image quality is poor or CMR is contraindicated or unavailable. Based on our experience and current literature, an optimal multimodality imaging approach should reserve EMB only for high-risk or inconclusive cases. Furthermore, this strategy offers complementary information, supporting clinical decisions and optimizing long-term outcomes.

## 1. Introduction

Eosinophilic myocarditis (EM) is a rare but potentially severe form of acute myocarditis (AM) characterized by eosinophilic myocardial infiltration, typically progressing to endocardial fibrotic remodeling with a high risk of intracavitary thrombus, a condition known as Loeffler cardiomyopathy. The true incidence of EM is difficult to determine because clinical presentation is nonspecific and endomyocardial biopsy (EMB) is not routinely performed during AM. Early diagnosis would enable prompt initiation of both etiologic and immunosuppressive therapies, which can significantly improve prognosis. The aim of this review is to explore how a multi-imaging approach can support an early diagnosis, reduce the need for EMB, stratify the risk and monitor the therapeutic response during the follow-up.

EM pathophysiology unfolds through four consecutive mechanisms: eosinophil hyperproduction, myocardial infiltration, degranulation and cardiac damage. Eosinophils are generated in the bone marrow in response to cytokine stimulation, primarily interleukin-5 (IL-5), which also inhibits eosinophil apoptosis in vitro [1]. Once released into the bloodstream, eosinophils migrate and accumulate in tissues releasing cytotoxic granules (direct damage) and induce chronic inflammation, vasculitis and fibrosis (indirect damage), which culminates in an organ injury. This pathological sequence is more likely when the absolute eosinophil count exceeds 1500/μL (hypereosinophilia), although it can also occur at lower levels.

## 2. Etiology

EM is a possible expression of hypereosinophilic syndrome (HES), a condition characterized by excessive and unregulated clonal expansion of eosinophils and organ damage. It is common practice to divide causes of HES into three categories: neoplastic (or primary), reactive (or secondary) and idiopathic.

In the neoplastic group, clonal expansion occurs in various myeloid neoplasms, such as acute or chronic eosinophilic leukemia, chronic myelomonocytic leukemia and systemic mast cell disease [2]. Malignant solid tumors, typically pulmonary adenocarcinomas, might also induce HES [3].

The reactive group includes helminthic infections (e.g., *Toxocara canis, Strongyloides, Schistosoma*) [4], allergies and atopic conditions (e.g., asthma, rhinitis, dermatitis) and drug reaction with eosinophilia and systemic symptoms (DRESS) syndrome induced after antibiotics, non-steroid anti-inflammatory, anticonvulsants or occasionally vaccination [5]. Additionally, reactive autoimmune diseases can cause eosinophilic syndrome, such as eosinophilic granulomatosis with polyangiitis, EGPA (or Churg–Strauss syndrome), a rare autoimmune vasculitis of small- to medium-sized blood vessels with clusters of inflammatory cells called granulomas leading to damage of various organs. EGPA seems to be secondary to immune system dysregulation and often develops in individuals with a history of asthma or allergic conditions.

Most cases of HES, however, do not have an identifiable underlying cause and are referred to as idiopathic HES.

## 3. Diagnosis

The suspicion of EM is primarily clinical, but the diagnosis requires a multimodality imaging approach integrated with laboratory findings and electrocardiography (ECG) evaluation. Nevertheless, definitive diagnosis relies on histological confirmation through EMB.

### 3.1. Clinical Features

**Figure 1:**
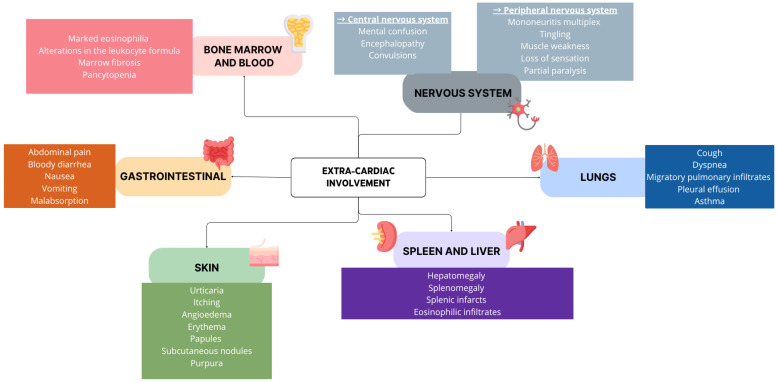
Possible extracardiac findings during hypereosinophilic syndrome (HES).

EM may affect middle-aged adults of both sexes equally [6]. According to the different possible etiologies, the clinical onset can range from subtle symptoms to acute heart failure (AHF) and, in severe cases, life-threatening fulminant myocarditis (FM) [7]; therefore, a high index of suspicion is crucial for timely and accurate diagnosis [8]. Patients could experience prodromal symptoms, such as flu-like illness, gastrointestinal issues, sore throat or respiratory infections; these symptoms can precede the acute phase by days or even weeks [9]. Fever is common (65%) [7], while dyspnea (19–49%) is less frequent and syncope (6%) is atypical [10]. Despite these variations, chest pain remains the most frequent symptom, occurring in 85–95% of cases [8]. In many cases, prodromal symptoms fail to alert patients, who often go to the emergency room only when AHF becomes evident—typically with dyspnea, fatigue and peripheral edema—or, in rarer cases of FM, hemodynamic instability develops in the context of cardiogenic shock (CS). Notably, due to the inflammatory nature of EM, patients are at a heightened risk for intracavitary thrombus and thromboembolic complications such as stroke or pulmonary embolism [11,12,13]. Moreover, as a systemic condition, extracardiac involvement is common (Figure 1).

### 3.2. Initial Work-Up

The initial work-up accounts for laboratory tests, chest x-ray and electrocardiography (ECG).

Laboratory evaluation should focus on detecting myocardial injury and systemic inflammation. Key tests include high-sensitivity troponins and creatine kinase-MB [14], although troponin levels correlate weakly with the degree of cardiac dysfunction [15]. Inflammatory markers such as C-reactive protein (CRP) are elevated in approximately 80–95% of cases [9]; similarly, erythrocyte sedimentation rate (ESR) is frequently increased, although this is less commonly used as a marker in emergency settings [16]. A complete blood count may show leukocytosis and eosinophilia [4], although not always present. Some patients may develop it during hospitalization, while in others, eosinophils may have already migrated to tissues, leading to transiently normal counts despite active marrow eosinopoiesis. Serial differential counts are therefore recommended to avoid missed or delayed diagnosis [17,18]. Autoantibody screening, including antinuclear antibodies (ANA), should be considered in patients with known or suspected autoimmune disease. In particular, EGPA diagnosis is based on hypereosinophilia, positive anti-neutrophil cytoplasmic antibodies (ANCA), imaging evidence of granulomas at chest X-ray (CXR) or at tissue biopsies and electromyography if nerves are involved [6]. Notably, the ANCA test is frequently negative in EM caused by EGPA [19,20]. Lastly, if a neoplastic or paraneoplastic eosinophilia is suspected, a tumor markers test should be considered [21].

Despite its nonspecificity, ECG remains an essential component in the initial assessment and risk stratification of EM. To note, ECG abnormalities are frequent (85%), and ST segment elevation—particularly in the inferior and lateral leads—is the most common finding, often mimicking acute myocardial infarction [7]. Diffuse ST segment changes, both elevations or depressions, and reduced QRS voltage are frequently observed in cases of pericardial involvement [22]. Repolarization abnormalities may manifest as negative, isoelectric, biphasic or triphasic T waves. Malignant arrhythmias, such as ventricular tachycardia or fibrillation, are fortunately uncommon and are associated with a poor prognosis [4]. Additional findings may include sinus tachycardia, bradycardia, atrioventricular block, bundle branch block or QRS prolongation [23].

CXR findings in EM are nonspecific. However, CXR is a valuable initial imaging modality and should be interpreted in conjunction with clinical presentation and other diagnostic modalities. It may reveal signs of left-side heart failure (e.g., cardiomegaly, pulmonary edema) and pericardial effusion, which may appear as a “water bottle” cardiac silhouette [24]. In case of suspected EGPA, CXR could show chest granulomas supporting the diagnosis [25]. However, this finding is not pathognomonic and is more accurately confirmed with echocardiography or CT imaging [22,24].

### 3.3. Echocardiography

Transthoracic echocardiography (TTE) is a non-invasive, rapid and easily available bedside examination that represents the first-line imaging modality in diagnostic EM.

**Figure 2:**
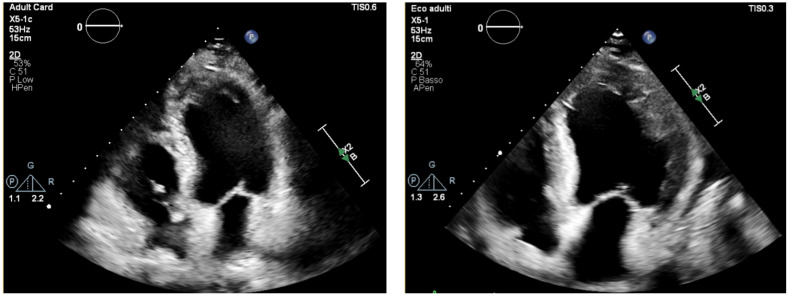
Transthoracic echocardiography, apical four-chamber view. Normal LV thickness and size (**left**), becoming pseudo-hypertrophic and hypokinetic after onset of eosinophilic myocarditis (**right**).

During the acute phase, TTE is important for assessing global cardiac function, through the left ventricular (LV) ejection fraction (EF). However, findings may be normal or slightly abnormal, such as hypokinesia, subendocardial hyperechogenicity, chamber dilation or increased wall thickness (pseudohypertrophy) [26] (Figure 2).

In the subsequent chronic phase, myocardial wall thickening and systolic dysfunction may persist, along with fibrotic obliteration of ventricular apices, intracavitary thrombus, atrial enlargement and valvular dysfunction.

**Figure 3:**
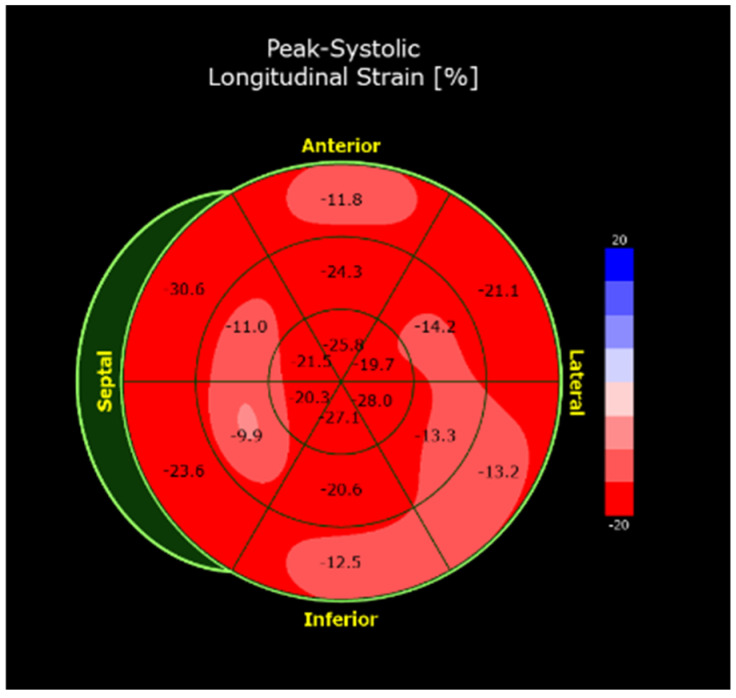
Bull’s-eye plot of GLS analysis shows a “reverse apical sparing” pattern, with lower longitudinal shortening values in some mid-basal segments (light red) and preserved values in apical segments (red). In clinical practice, values less negative than -16% are considered reduced [41].

Global longitudinal speckle (GLS), assessed by speckle tracking echocardiography, is useful for assessing myocardial deformation and identifying early systolic dysfunction, even when standard LVEF is still preserved [27]. In the chronic phase, a pathological pattern predominantly involving the apex, known as “reverse apical sparing”, may be observed [28] (Figure 3). This can also be observed in hypertrophic cardiomyopathy; GLS values are typically preserved in this setting [29]. Left ventricular mechanical dispersion (LVMD) is an emerging parameter that strongly correlates with the risk of ventricular arrhythmias and overall prognosis. It may outperform both GLS and LVEF in terms of predictive value. However, it has not yet been studied in the context of AM and further research is required to define clinically meaningful cutoff values [30,31,32]. Regarding diastolic dysfunction, elevated filling pressures may predict progression toward restrictive cardiomyopathy [33,34,35]. For the detection of intracavitary thrombi, particularly at the LV apex, specific intravenous ultrasound contrast agents can be useful. These agents are also beneficial for better characterization of ventricular function and for differential diagnosis with apical hypertrophic cardiomyopathy [28,36]. Regurgitation of the atrioventricular valves is a common finding, likely secondary to inflammatory damage and altered cardiac hemodynamics. Specifically, mitral regurgitation derives from chronic restriction of posterior leaflet motion [37,38] and tricuspid regurgitation may result from direct inflammatory injury or be a consequence of pressure overload due to left-sided valvular disease [37,38]. Severe mitral stenosis secondary to EGPA has also been reported, with a positive response to pharmacological treatment [39]. In case of persistent doubts regarding valvular anatomy and intracavitary thrombus, transesophageal echocardiography can be helpful [35,40].

### 3.4. Cardiac Magnetic Resonance

CMR is the non-invasive gold standard for diagnosing myocarditis, according to the 2018 Lake Louise criteria, with a sensitivity of 87.5% and specificity of 96.2% [42,43]. The integration of CMR and highly sensitive troponin further improved non-invasive diagnostic accuracy [42], thus increasingly reserving EMB for high-risk cases. CMR uniquely provides tissue characterization similar to a virtual biopsy, while also offering high spatial resolution and accurate assessment of cardiac function. It is also valuable for identifying intracavitary thrombotic formations, guiding potential biopsy procedures, and ruling out coronary artery disease (CAD). Four weeks from symptom onset, myocardial edema tends to regress [44], so experts recommend performing CMR within 2–3 weeks from symptom onset, depending on hemodynamic stability [6]. An ideal CMR protocol for suspected EM should include the following sequences: (a) cine Steady-State Free Precession (cine-SSFP) for atrial dimensions and biventricular assessment in terms of global and regional systolic function, chamber size, wall thickness and myocardial mass; (b) native and post contrast T1 mapping, for quantitative calculation of the extracellular volume (ECV); (c) native T2 mapping, for the identification and quantification of myocardial edema; (d) Late Gadolinium Enhancement (LGE), for the detection of myocardial edema and fibrosis/necrosis. It is noteworthy that if native T1 and T2 mapping are not available, T2-weighted Short Tau Inversion Recovery (T2w-STIR) sequences can be performed for visual detection of myocardial edema.

In acute EM, diffuse myocardial edema is commonly observed. This condition may lead to apparent hypertrophy and increased signal intensity on both T1w and T2w sequences, accompanied by elevated native T1 and T2 mapping values, and consequently an increased ECV. According to our experience, the presence of LGE may be diffuse and without a specific pattern, reflecting the presence of an ongoing myocardial edema [45,46,47]. Notably, cases of acute necrotizing forms of EM have been reported with no detectable abnormalities on CMR, except for a mild pericardial effusion [48,49].

**Figure 4:**
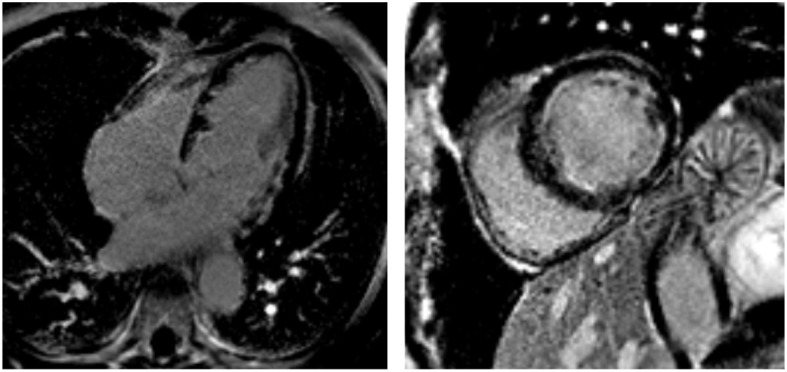
Loeffler cardiomyopathy. LGE sequences performed 5 months after the onset of eosinophilic myocarditis show a circumferential pattern of subendocardial fibrosis (hyperintense areas) in the apical four-chamber view (**left**) and mid-ventricular short-axis view (**right**). The subendocardial fibrosis does not follow a coronary distribution and produces a “V-shaped” appearance of the apical ventricles in the apical four-chamber view.

**Figure 5:**
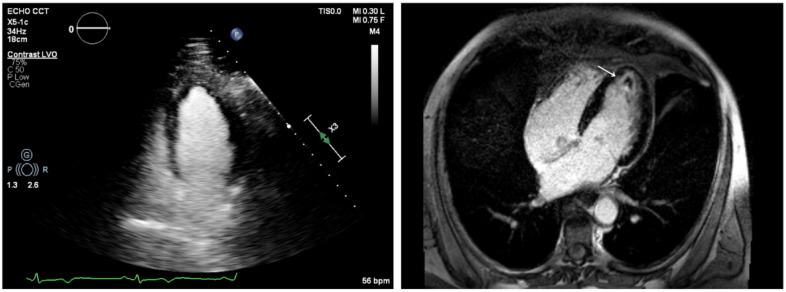
Patient with Loeffler cardiomyopathy and high suspicion of intracavitary thrombosis due to a transient ischemic attack (TIA). Contrast-enhanced echocardiography does not show intracavitary thrombus in the left ventricle (LV) (**left panel**; the green line represents the simultaneous electrocardiographic [ECG] recording), whereas CMR reveals a small apical thrombus in the LV apex (white arrow, **right panel**).

In subacute and chronic EM phases, the presence of myocardial edema is variable, while LGE typically reveals a circumferential pattern of subendocardial fibrosis [50,51,52,53], occasionally involving both ventricles (“V-shaped” aspect of the apical ventricles) [54], and clearly not following a coronary distribution. This fibrotic progression, known as Loeffler cardiomyopathy (Figure 4), is particularly common in EGPA-related EM [4]. At this stage, it is crucial to rule out intraventricular thrombus, not always detectable by contrast echocardiography (Figure 5). Moreover, a correlation has been observed between the CMR pattern and the histopathological subtype. Notably, necrotizing EM is associated with the presence of subendocardial LGE in a greater number of LV segments, and the LGE mass is approximately double compared to forms without myocardial necrosis [55].

### 3.5. Cardiac CT

Cardiac computed tomography (CT) can play a role in the diagnostic work-up of EM, especially in patients who cannot undergo CMR, despite its associated radiation exposure and iodine contrast administration. EM onset could mimic an acute coronary syndrome, the cardiac CT rules out CAD in patients with low pretest clinical likelihood, thereby avoiding invasive coronary angiography [37,56,57,58], allowing the evaluation of coronary anatomy variants (e.g., anomalous origin, myocardial bridge), atherosclerosis and myocardial perfusion, distinguishing ischemic vs. non-ischemic origins of LV dysfunction [59,60]. Cardiac CT can also serve as a validated alternative to ETT and CMR for the evaluation of cardiac volumes, dimensions and bi-ventricular EF when echocardiographic image quality is suboptimal and CMR is either contraindicated (e.g., non-MRI-compatible devices or severe claustrophobia) or unavailable [61,62,63,64]. Finally, cardiac CT can detect LV thrombus more effectively than echocardiography; it appears as a crescent-shaped filling defect with hypoattenuation (<65 Hounsfield units) [65,66].

### 3.6. [^18^F]FDG Positron Emission Tomography

Positron emission tomography (PET) is not routinely used in EM or AM in general. Hybrid techniques such as 2-[^18^F]fluorodeoxyglucose ([^18^F]FDG) PET/CT or PET/MRI can serve as valid non-invasive alternatives to CMR for diagnosing myocarditis. These techniques combine the detection of metabolically active inflammation with detailed anatomical information [67]. The diagnostic accuracy of PET in this context is high when compared to EMB [68]. However, its effectiveness depends on several factors, including the timing of imaging, which should ideally be performed within 14 days of symptom onset, with adequate dietary preparation with a glucose-free regimen maintained for at least 12 h, and corticosteroid therapy withdrawal in the 72 h preceding the scan. Specifically, inflamed myocardial tissue typically demonstrates decreased perfusion and increased [^18^F]-FDG uptake, due to the accumulation of activated leukocytes, especially macrophages, which express high levels of glucose transporters. This leads to rapid and localized tracer accumulation at sites of inflammation [69]. Moreover, [^18^F]FDG PET can be a valuable guide for targeted EMB, detection of inflammatory processes in other organs in patients with underlying systemic autoimmune diseases, and for monitoring response to immunosuppressive therapy [70,71].

While [18F]FDG PET is currently the primary molecular imaging method for myocardial inflammation, its clinical application is limited by physiological myocardial tracer uptake and its tendency for non-specific uptake in various cardiac diseases. New PET radiotracers targeting inflammatory immune cells or distinct molecular pathways, compared to [18F]FDG, may provide better visualization of the underlying mechanisms in inflammatory cardiac diseases, including EM [72]. However, the current literature on PET/CT or PET/MRI with different radiopharmaceuticals in EM is limited to case reports [73,74,75,76] and thus, only very low-quality evidence supports the use of PET in EM.

### 3.7. Endomyocardial Biopsy

EMB remains the gold standard for the diagnosis of myocarditis [56], particularly useful when the clinical presentation and other diagnostic methods fail to provide a definitive diagnosis [77]. The invasive nature of EMB and its potential complications, reported in rates ranging from <1 to 6% depending on the clinical scenario and on operator’s experience [78], discourage the routine use of EMB in the management of uncomplicated myocarditis. Potential complications of EMB include arrhythmias, bleeding and myocardial perforation [78]. EMB should be reserved for AM cases with undetermined etiology, and in the case of FM, to provide a timely and definitive diagnosis and guide the appropriate therapy [79]. The decision to perform a right or left ventricular biopsy should be guided by clinical presentation and ventricular involvement; advanced imaging, such as CMR, but also PET, might help define target areas for EMB [80]. However, it should be noted that LV EMB is associated with an almost two-fold increased risk of complications compared with RV or EMB and should only be considered when inflammation affects exclusively the LV [78]. The sensitivity of EMB is relatively low due to sampling error, as biopsies may miss inflamed areas, particularly in focal myocarditis [81]. To enhance diagnostic accuracy and reduce sampling error, EMB should be performed early in the disease course (e.g., prior to eventual anti-inflammatory therapies), with the acquisition of multiple tissue specimens [81]. A minimum of three samples, each measuring 1–2 mm, should be collected from either the RV or LV and immediately fixed in 10% buffered formalin at room temperature for histological evaluation by light microscopy. Diagnostic interpretation should follow the Marburg criteria, which define active myocarditis by the presence of ≥14 leukocytes/mm^2^—including up to 4 monocytes/mm^2^—with ≥7 CD3-positive T lymphocytes/mm^2^, along with evidence of myocardial necrosis or degeneration not characteristic of ischemic damage [56,82]. To improve the diagnostic yield of immunohistochemical analysis, the use of a broad panel of monoclonal and polyclonal antibodies is recommended [83]. In EM, histology shows abundant eosinophilic infiltration without giant cells or granulomas. Although EMB remains limited in the overall workup of suspected myocarditis, its role specifically for EM might be more relevant due to the often acute presentation of this form and to the absence of a pathognomonic aspect in non-invasive imaging.

The main clinical indications for EMB in suspected eosinophilic myocarditis, along with the role of imaging in improving diagnostic yield and biopsy guidance, are summarized in Table 1.

## 4. Treatment and Follow-Up

### 4.1. Fulminant and Acute Non-Fulminant Myocarditis

The two possible cardiac presentations of EM are acute non-fulminant and fulminant myocarditis (FM).

FM with CS represents the most severe form of presentation, with a reported mortality rate or requirement for heart transplantation (HTx) of 26.3% at 60 days and 37.3% at 1 year [84]. Although there is no standardized protocol, a recent expert consensus statement on the management of CS [85] highlights that continuous invasive hemodynamic monitoring within the first 24 h is essential to assess CS severity and appropriately titrate pharmacological and/or mechanical support. The goal of vasoactive pharmacological support is to improve cardiac output and maintain adequate end-organ perfusion. Although no agent has demonstrated clear superiority, norepinephrine is generally considered the first-line inopressor, while dobutamine is commonly used as an inodilator. Calcium sensitizers such as levosimendan can also be beneficial, although their use may be limited by arterial hypotension or renal failure. In the first 24 h, if signs of hypoperfusion persist or worsen despite pharmacological support, an early intervention with temporary Mechanical Circulatory Support (t-MCS) should be considered. The aim is to improve organ perfusion, unload the ventricles, reduce pharmacological support, facilitate myocardial recovery or act as a bridge to a left ventricular assist device (LVAD) or HTx.

Acute non-fulminant EM is characterized by AHF onset and its management follows the 2021 ESC Guidelines for the diagnosis and treatment of acute and chronic heart failure [86]. When present, congestion/fluid overload should be managed with intravenous loop diuretics. In cases of persistent congestion and reduced urine output (<100–150 mL/h after 6 h), renal replacement therapy with ultrafiltration may become necessary. In patients with heart failure with reduced ejection fraction (HFrEF), empirical initiation and titration of the four cornerstone therapies for heart failure should be performed, although there is a lack of evidence supporting their mortality benefit in cases of heart failure secondary to AM.

### 4.2. Thromboembolic Complications

Intracardiac thrombosis, particularly at the LV apex, is a frequent finding in EM, especially in forms associated with hypereosinophilia and EGPA [4]. Oral anticoagulant (OAC) therapy is recommended for a minimum duration of three months. According to a recent meta-analysis [87], direct oral anticoagulants (DOACs) represent a valid alternative to vitamin K antagonists (VKAs), particularly when international normalized ratio (INR) monitoring is challenging or therapeutic targets are not consistently achieved. After the initial treatment period, preferably CMR but also TTE or contrast TTE, it is essential to evaluate thrombus resolution and determine whether OAC therapy can be discontinued. Regardless of thrombus regression, prolonged or indefinite anticoagulation should be considered if persistent apical akinesia or underlying prothrombotic and pro-inflammatory conditions are present.

### 4.3. Inflammatory Disease

Treatment of EM depends on the underlying cause of HES. In cases related to clonal myeloid disorders, tyrosine kinase inhibitors are employed, whereas parasitic infections require appropriate anthelmintic therapy. In hypersensitivity reactions, identification and withdrawal of the offending agent are essential. When EM is associated with EGPA or in the case of idiopathic HES, immunosuppressive therapy represents the mainstay of treatment, with corticosteroids being the first-line agents, showing efficacy in up to 85% of cases. Management strategies are largely based on expert consensus and are often adapted from established protocols for other inflammatory cardiomyopathies [88]. Additionally, intravenous immunoglobulin (IVIG) has been used as a therapeutic option in immune-mediated or FM cases [89]. In patients who are refractory to corticosteroids or experience significant side effects, additional immunosuppressive agents such as cyclophosphamide, methotrexate or azathioprine may be considered. Furthermore, biologic agents such as mepolizumab and benralizumab, targeting IL-5 and its receptor, respectively, have shown promising results. However, evidence supporting their efficacy is currently limited to case reports, small case series or extrapolations from studies on other eosinophilic disorders, and their impact on long-term outcomes remains unproven [90]. Similarly, the potential clinical benefit of combination immunosuppressive regimens—including glucocorticoids, azathioprine or cyclosporine [91]—remains uncertain, as evidence is limited to observational studies not specific to EM and is further confounded by the high rate of spontaneous recovery. An overview of these treatment strategies and the available evidence is provided in Table 2 [92].

### 4.4. Multimodality Imaging in the Follow-Up

The choice of imaging modality during follow-up in EM is influenced by the disease phase, clinical status, and specific monitoring objectives [93].

TTE remains a first-line tool due to its accessibility and capacity for repeated and real-time evaluation of cardiac function. It is especially useful for monitoring LV systolic performance and detecting evolution toward restrictive cardiomyopathy with fibrotic involvement of myocardial and valvular structures. Studies report a median LVEF at presentation of 35% (IQR 25–50%), with recovery by discharge, except in EGPA-associated forms where median pre-discharge LVEF remains at 40% (IQR 31–49%) [4,37]. Tissue Doppler Imaging (TDI) offers additional prognostic value. Reduced S′ and e′ velocities may persist despite the normalization of LVEF, reflecting ongoing subclinical systolic and diastolic dysfunction. Echocardiography is also essential for monitoring pericardial effusion and intracavitary thrombus [37,94].

Based on our clinical experience and evidence from the literature [95,96,97,98], a structured imaging follow-up strategy is advisable. After discharge, TTE should be repeated at 4–6 weeks to monitor functional and structural recovery, while CMR should be performed at 2–3 months to assess the transition from edema to fibrotic remodeling; if CMR is unavailable or contraindicated and residual inflammation is still suspected, [^18^F]-FDG PET is a valid alternative. To reassess intracavitary thrombus, both CMR and echocardiography should be repeated after at least three months of OAC.

## 5. Conclusions

EM is a rare and potentially fatal form of AM. A multimodality approach enables early diagnosis, risk stratification and monitoring of the response to anti-inflammatory therapy, thereby potentially reserving EMB only for high-risk patients or inconclusive imaging findings.

## Figures and Tables

**Table 1:** Clinical indications for endomyocardial biopsy in suspected EM and the role of imaging in guiding biopsy.

CLINICAL SCENARIO	EMB RATIONALE	IMAGING MODALITY	IMAGING ROLE IN BIOPSY
**Fulminant myocarditis with shock**	Rapid diagnosi sto guide immunosoppressive/antiviral therapy. Note: imaging may be not always feasible in unstable patient.	CMR, [18F]FDG, PET/CT	Identify areas with highest oedema or tracer uptake if patients’ conditions allow imaging
**Unclear diagnosis after imaging**	Histological confirmation (e.g., EM vs. other)	CMR	Locate subendocardial LGE or diffuse edema to target biopsy site
**No reponse to therapy**	Explore alternative or coexisting causes	PET	Confirm persistent inflammation and guide sampling
**Isolated LV involvement**	Consider LV biopsy (with caution)	CMR	Confirm lack of RV involvement and focus on LV lesions for biopsy guidance
**Focal myocarditis**	Increase diagnostic yield	CMR PET	Avoid inaffected areas, reduce false negatives
**Systemic disease with cardiac involvement (EGPA, HES)**	Confism eosinophilic infiltration	PET/TC CMR	Define cardiac and extracardiac disease pattern, guide EMB to active sites

**Table 2:** Anti-inflammatory treatment strategies in EM.

LINE	THERAPY	DOSE	DURATION
**FIRST LINE**	Methylprednisone (IV pulse therapy) Prednisone (oral)	500 mg to 1 g/day 1 mg/kg/day Max: 60–80 mg/day	3–5 days Several weeks, with a slow taper over 3–6 months
**ADJUNCTIVE OR RESCUE THERAPY**	Intravenous immunoglobulin (IVIG)	2 g/kg total dose, typically given as: 400 mg/kg/day 1 g/kg/day	For 5 days For 2 days
**IF STEROID-REFRACTORY**	Azathioprine Mycophenolate mofetii Cyclophosphamide	1–2 mg/kg 1–2 g 1–2 mg/kg orally Or IV pulse (500–1000 mg/m^2^)	Daily Daily Daily monthly

## Data Availability

No new data were created or analyzed in this study.

## Abbreviations

The following abbreviations are used in this manuscript:

[18F]FDG	[18F]fluorodeoxyglucose
AC	acute myocarditis
AHF	acute heart failure
ANA	antinuclear antibodies
ANCA	anti-neutrophil cytoplasmic antibodies
CAD	coronary artery disease
cine-SSFP	cine steady-state free precession
CMR	cardiac magnetic resonance
CRP	C-reactive protein
CS	cardiogenic shock
CT	computed tomography
CXR	chest X-ray
DOACs	direct oral anticoagulants
DRESS	drug reaction with eosinophilia and systemic symptoms
ECG	electrocardiography
ECV	extracellular volume
EF	ejection fraction
EGPA	eosinophilic granulomatosis with polyangiitis
EM	eosinophilic myocarditis
EMB	endomyocardial biopsy
ESR	erythrocyte sedimentation rate
FM	fulminant myocarditis
GLS	global longitudinal speckle
HES	hypereosinophilic syndrome
HFrEF	heart failure with reduced ejection fraction
HTx	heart transplantation
IL-5	interleukin-5
INR	international normalized ratio
IVIG	intravenous immunoglobulin
LGE	late gadolinium enhancement
LV	left ventricular
LVAD	left ventricular assist device
LVEF	left ventricular ejection fraction
LVMD	left ventricular mechanical dispersion
OAC	oral anticoagulant
PET	positron emission tomography
PET/CT	positron emission tomography/computed tomography
t-MCS	temporary mechanical circulatory support
T2w-STIR	T2-weighted short tau inversion recovery
TDI	tissue doppler imaging
TTE	transthoracic echocardiography
VKAs	vitamin K antagonists
